# An Experimental Essay on the Relative Physiological and Medicinal Properties of Iodine and Its Compounds

**Published:** 1839-01-01

**Authors:** 


### AN EXPERIMENTAL ESSAY ON THE RELATIVE PHYSIOLOGICAL AND MEDICINAL PROPERTIES OF IODINE AND ITS COMPOUNDS; BEING THE HARVEIAN PRIZE DISSERTATION FOR 1837. By *Charles Cogswell*, A.B. M.D., &c. Octavo, pp. 179. Edinb. 1837.

WE owe Dr. Cogswell an apology for not having noticed his Essay sooner. Our apparent neglect of it has arisen from accidental circumstances solely, and not from an unfavourable opinion as to its merits. On the contrary, we regard this first production of Dr. Cogswell's pen, as at once an evidence of his past industry, and an omen of his future professional success.

Since the first introduction of Iodine into medicine, we have not failed, from time to time, to make our readers acquainted with all the really important observations that have been published respecting it. In our number for January, 1832, we inserted a minute and lengthened analysis of Dr. O'Shaughnessy's Translation of M. Lugol's work; and to this we refer our readers for many observations of a very valuable nature.

Dr. Cogswell, after discussing the history both of burnt sponge and iodine, details briefly the chemical properties of the latter, and then proceeds to treat of its "physiological action." Dissatisfied with an experiment of Orfila, which goes to prove that the presence of iodine in the cellular tissue of animals does not produce any injurious constitutional effects, he repeated it, with a result apparently different. We can see no good whatever in the practice of such barbarities. The local lesion must, of itself, produce more or less constitutional irritation, and cannot fail therefore to prove a perplexing element in the calculation and estimate of results. It might fairly be argued from Dr. C.'s own experiment, that as the constitutional symptoms did not display themselves until a fortnight had elapsed, they were attributable solely to the "large abscess" created by the local action of the iodine. Dr. C. himself notices this source of fallacy, and states it as a sufficient reason for not repeating the experiment.

The physiological effects of iodine are described as they are produced by large, or by small and repeated doses of the substance. This very apposite and convenient method is adopted after the example of Dr. Christison. It would appear from some well-authenticated observations, that iodine may be taken in very large quantities without producing poisonous effects. As it forms an insoluble compound with albumen, food containing a large proportion of that substance must very much modify its operation. But though in some cases it would appear to have proved an inert agent, in many others it has been known to give rise to very violent symptoms. Nor can this be wondered at by any one who has witnessed the effects of its application to the skin. But there are some men in our profession who make it their object to discover, not how much of any medicine may be sufficient to effect a desired object, but how much a man may take without being killed by it. These empirics (we use the word in the ancient sense) have certainly proved, that the mucous membrane of the stomach and bowels can bear more than, but for their temerity, we should ever have supposed possible.

Dr. Cogswell injected two drachms of the tincture of iodine into the jugular vein of each of two dogs. Both died. In a similar experiment with a third dog, he injected only one drachm, and the unfortunate brute recovered on the third day. We can glean no useful results from these unfeeling experiments. Those who practise them on a large scale, must, we have often thought, approximate in nature much more nearly than ordinary men to the Yahoos of Houyhnhnm land, who were remarkable both for their filthiness and their cruel disposition.*

In small doses frequently repeated, iodine acts as an excitant of the whole system, but primarily and more especially does it affect the digestive mucous membrane. When continued injudiciously, it often, according to our own experience, gives rise to a peculiar kind of fever, characterized by breathlessness, palpitation, a very quick and weak pulse, urgent thirst, and a sense of extreme weakness, which last symptom is that of which the patient most complains. The symptoms in fact resemble very closely those of *mercurial erethism*. They may be removed in a few days by discontinuing the use of the iodine, and prescribing small doses of ammonia.

After discussing the question, " is iodine a cumulative medicine," and failing to come to any positive conclusion thereupon, Dr. Cogswell treats of its different formulæ and the manner of their application; and then proceeds to consider the several diseases in which iodine has been administered with advantage. His authorities are selected with industry and judgment, and he has the rare merit of not being prolix. To *analyse* this part of the work, which is itself an analysis of almost all that has been written on the subject, would be difficult; and as it does not contain any new observations, we pass it over with this remark, that we are firmly convinced the general curative properties of iodine have been greatly exaggerated. As to its efficacy in what are called " nervous diseases," we believe it gene-

---

* *Suum cuique* is a maxim which we approve of so highly, that we have often had it in our mind to write a book, expressly on purpose to shew how many pretended modern discoveries in medicine may be traced back to the older authors. The above allusion to Gulliver's Travels reminds us that Dr. O'Beirne (who needed as little as most men to claim another's merit) has never acknowledged (which he ought in justice to have done) that he borrowed his plan of defæcation from one of the professors in the grand academy of Lagado, whose mode of curing a colic is thus described. "He had a large pair of bellows, with a long slender muzzle of ivory; *this he conveyed eight inches up the anus, and drawing in the wind, he affirmed he could make the guts as lank as a dried bladder*. But when the disease was more stubborn and violent, he let in the muzzle while the bellows were full of wind, which he discharged into the body of the patient; then withdrew the instrument to replenish it, clapping his thumb strongly against the orifice of the fundament; and this being repeated three or four times, the adventitious wind would rush out, bringing the noxious along with it, (like water put into a pump,) and the patient recover."—*Voyage to Laputa*, Chap. V.

We have given here at full length the celebrated professor's method of cure, but Dr. O'Beirne has adopted only the former process, being most probably afraid of putting in practice the latter, and not, we must own, without reason; for we are told that the dog on which it was tried " died on the spot." At the same time (it is due to our readers to add) the excellent historian from whom we quote, asserts plainly, that he " *could not discern any effect from the former process*."—*Rev.*

rally does more harm than good. Even in scrofula, we doubt whether, as an internal agent, it effects nearly so much good as it is generally thought to do. Its external application as a counter-irritant is under many circumstances productive of benefit. But routine practitioners, with whom iodine, in its several forms, is a sort of panacea, very often (we speak from personal observation) apply it to joints affected with acute synovial inflammation. In fact, iodine is now so universally, so thoughtlessly, and indiscriminately employed in the treatment of almost all diseases, that, by a fate common to all over-praised remedies, it must needs fall, before many years shall have passed, into even unmerited neglect.

We have thus taken a survey, as it were, of 84 pages of Dr. Cogswell's Treatise. There still remain an equal number which are occupied in treating of the several preparations of iodine. These are severally considered, first, with reference to their physiological action, and, secondly, as regards their medicinal effects. The section which treats of the iodide of potassium, or the hydriodate of potash as it is generally called, extends from the 85th to the 117th page, both inclusive. It contains an excellent summary of all that is known on the subject, and is well worthy the attention of the reader.

There is in this part of the work a coloured plate, which represents the inflammatory results produced in the stomach of a rabbit, by the injection of a drachm of hydriodate of potash, dissolved in two drachms of water.

While on the subject of the hydriodate of potass, we may state that we have found it, in combination with tincture of iodine, diluted with water, prove a most efficient application in more than one of the worst forms of porrigo.

Of the remaining compounds of iodine we shall notice only one—the iodide of zinc, and that too solely for the purpose of remarking, that during the last six years we have occasionally employed it, with apparent advantage, in a variety of scrofulous affections. We were first led to do so by having noticed the marked efficacy of sulphate of zinc dissolved in distilled water, when used as an injection for scrofulous sinuses. It was an easy and a natural step to come to the resolution of trying zinc and iodine in combination. We have usually begun with one grain of the compound, dissolved in infusion of quassia, for a dose, and have never gone beyond three grains. But we dare say that if the medicine should ever come into general use, some very clever person will discover that a patient may take a drachm or more—without being killed by it.

During the last two years we have been in the habit of employing a strong solution of the iodide of zinc, as an application to the tonsillary glands, when affected with chronic enlargement; and we can recommend it to our readers as the best *local* remedy we know for that most obstinate complaint.

There is attached to Dr. Cogswell's work an Appendix of nine closely-printed pages, which contain several observations worthy the notice of the reader.

Our opinion of Dr. Cogswell's Treatise has been more than once expressed in the foregoing pages. In such a book, written, as it would appear, by one who had just completed his university studies, much original matter was not to be expected. But judicious selection, clear arrangement, and a just proportioning of attention to the several parts of the subject—these it was not too much to look for in a work which had obtained the prize of the Harveian Society; nor have we looked for them in vain.

The *style* of a medical writer is only a matter of secondary consideration, unless when it is so obscure as to make the sense doubtful to readers of ordinary capacity. But in the case of young authors, it seems desirable to attach more importance to it, so that by early correction of any errors into which they may fall, they may come at length to write with both clearness and accuracy. We have no particular fault to find with Dr. Cogswell's style, except that the sentences are sometimes too long or too complicated. As an instance of the latter, we quote, almost at random, one from the Introduction, (page 12.)

"But, as we hinted above, and the fact will only become too manifest in the subsequent pages, from the experience of all parties, himself included, without paying the most scrupulous attention to the chemical habitudes of iodine, we shall constantly be liable to form erroneous or imperfect conclusions."

It is easy to perceive how the above sentence might have been made more intelligible, by separating in a more marked manner the parenthetical clause; but it would have been still better to have constructed it in a different and more simple manner. We make this piece of criticism (which, after all, is not worth much) in a good spirit, and we beg to take our leave of Dr. Cogswell by wishing him that success in his profession which his industry and talents seem to deserve.

